# Cancer Immunotherapy: The Checkpoint between Chronic Colitis and Colorectal Cancer

**DOI:** 10.3390/cancers14246131

**Published:** 2022-12-12

**Authors:** Ramya Ephraim, Jack Feehan, Sarah Fraser, Kulmira Nurgali, Vasso Apostolopoulos

**Affiliations:** 1 Institute for Health and Sport, Victoria University, Melbourne, VIC 3030, Australia; 2 Australian Institute for Musculoskeletal Science, Melbourne, VIC 3021, Australia

**Keywords:** checkpoint, checkpoint inhibitors, checkpoint markers, TIM-3, PD-1, PD-L1, CTLA-4, LAG-3, IDO, Siglec, inflammatory bowel disease, colorectal cancer, inflammation

## Abstract

**Simple Summary:**

Inflammatory bowel disease (IBD) affects the colon and is divided in two main pathologies, ulcerative colitis and Crohn’s disease. It is characterised by inflammation, which is managed by anti-inflammatory treatments, however, in the long term they lose effectiveness. Chronic inflammation/chronic colitis pre-disposes the person to increased risk of colorectal cancer (CRC). Checkpoint markers has revolutionised immunotherapeutic treatments especially in colorectal cancer. Here, we present different checkpoint inhibitors and their role in IBD and CRC.

**Abstract:**

Inflammatory Bowel Disease (IBD) is a group of diseases that cause intestinal inflammation and lesions because of an abnormal immune response to host gut microflora. Corticosteroids, anti-inflammatories, and antibiotics are often used to reduce non-specific inflammation and relapse rates; however, such treatments are ineffective over time. Patients with chronic colitis are more susceptible to developing colorectal cancer, especially those with a longer duration of colitis. There is often a limit in using chemotherapy due to side effects, leading to reduced efficacy, leaving an urgent need to improve treatments and identify new therapeutic targets. Cancer immunotherapy has made significant advances in recent years and is mainly categorized as cancer vaccines, adoptive cellular immunotherapy, or immune checkpoint blockade therapies. Checkpoint markers are expressed on cancer cells to evade the immune system, and as a result checkpoint inhibitors have transformed cancer treatment in the last 5–10 years. Immune checkpoint inhibitors have produced long-lasting clinical responses in both single and combination therapies. *Winnie* mice are a viable model of spontaneous chronic colitis with immune responses like human IBD. Determining the expression levels of checkpoint markers in tissues from these mice will provide insights into disease initiation, progression, and cancer. Such information will lead to identification of novel checkpoint markers and the development of treatments with or without immune checkpoint inhibitors or vaccines to slow or stop disease progression.

## 1. Introduction

Inflammatory bowel disease (IBD), also known as chronic colitis, is an idiopathic disease that causes intestinal inflammation and lesions due to an altered immune response to host gut microflora [1,2]. Approximately 80,000 individuals in Australia are living with IBD, with 5500 new cases diagnosed annually; globally this number equates to 6.8 million individuals with females being more affected than males. Available treatments for IBD in surgery, corticosteroids, anti-inflammatories (aminosalicylates, balsalazide and olsalazine), antibiotics and immune suppressors (azathioprine, methotrexate, mercaptopurine), all of which aim to reduce non-specific inflammation to decrease relapse rates. Such treatments, however, are ineffective in the long term [3]. Gradual development of inflammation causes oedema in the intestinal wall, ulceration, and long term can lead to colorectal cancer (CRC) [4]. There is often a limit in using chemotherapy due to side-effects leading to reduced efficacy, making evaluation of improved treatments, as well as new targets and mechanisms critical.

Inflammation is a core feature of several diseases including IBD. Chronic inflammatory diseases frequently result in the development of poorly regulated cellular processes that may lead to cancer [5]. Colitis-associated cancer (CAC) is a type of CRC that develops following a long period with IBD. The link between inflammation as a potential cause in CRC is being studied. Crohn’s disease (CD) is associated with a greater risk of developing CAC by up to 8.3% and ulcerative colitis (UC) by up to 33.2% when compared to the non-IBD affected population’s risk of developing CRC.

Cancer immunotherapy dates back to 1891, when William Coley, the father of immunotherapy, attempted to use the immune system to treat cancer after discovering that combinations of live and inactivated *Streptococcus pyogenes* and *Serratia marcescens* were able to induce tumour regression in patients with sarcoma [6,7]. Since then, it has evolved into a novel and effective method of treating cancer by enhancing the immune system instead of directly targeting tumour cells with chemotherapeutics [8,9]. Immunotherapies can be broadly classified into cancer vaccines [10,11,12], adoptive cellular immunotherapy, or checkpoint inhibitor therapy [13,14]. Checkpoint markers are present on cancer cells to evade the immune system, and immune checkpoint inhibitors (ICIs) operate by inhibiting these checkpoint proteins/markers, allowing the immune system to destroy cancer cells. In recent years, the use of ICIs has improved cancer treatment [15,16,17,18,19,20,21].

## 2. Inflammatory Bowel Disease

IBD is a chronic condition with 2 major pathologies, UC and CD [2]. The typical clinical symptoms for IBD include gut hypersensitivity and abdominal pain which are associated with chronic diarrhea and rectal bleeding [22]. CD is mainly characterised by severe chronic inflammation expressed as trans-mural skip lesions across the intestinal tract and UC exhibits continuous mucosal and submucosal inflammation extending from the rectum to the colon [23]. IBD’s immune characteristics are a result of aberrant responses of the innate and adaptive immune systems [24]. Approximately 95% of UC patients have inflammation in the rectum, with 25% having inflammation restricted to the rectum [25]. Long-term management of IBD is challenging due to toxic long-term effects of therapies or unresponsiveness in patients [26]. IBD is most common between the second and third decades of life, with another high point between the ages of 60 and 70 [24]. There is a significant increased risk of new mental illness postpartum among women with IBD, specifically in the presence of CD [27], with the rate of depression and anxiety being highest during active disease states [28]. Of relevance, it was recently shown that inflammation in the intestines caused by an acute dose of methamphetamine causes leaky gut syndrome, systemic inflammation, inflammation in the brain and mood disorders such as anxiety [29,30,31]. Gut inflammation and changes in behaviour are closely related. In recent years, a range of IBD therapeutic drugs have emerged that include sulfasalazine, azathioprine, corticosteroids, classical immunosuppressive agents, and anti-tumour necrosis factor (TNF)-α antibodies [32].

### Mouse Models

Mouse models of IBD and CRC are widely used to help in the understanding of how living tissues function and the biology of underlying gut diseases [33]. Computer models and intestinal cell cultures are also used to study gut disorders; however, such models cannot replicate complicated interactions that exist in the whole digestive system, especially in the gut where the extremely important interactions between the mucus and the gut microbiome take place. Although cell lines are effective models for studies and are commonly used to understand factors released by cancer cells and their receptors, they cannot be used for studies on tumour growth and metastatic spread of cancer [34]. In mice models, acute and chronic colitis are mediated intrarectally by administering 2,4,6-trinitro benzene sulfonic acid (TNBS), oxazolone, which induces a T-cells against hapten modified autologous proteins [35]. In comparison to dinitrobenzene sulfonic acid (DNBS), TNBS is considered toxic due to its highly oxidative properties, which can lead to an explosion when in contact with bases like sodium and potassium hydroxide [36]. The most used chemically induced model of intestinal inflammation is dextran sulphate sodium (DSS). Mice are fed DSS-enriched water for several days, which appears to be especially harmful to colonic epithelial cells of the basal crypts [35]. Colitis in these animal models result from injury repair like UC, but it must be controlled to prevent differences in DSS concentration and irregular water uptake by the animals; this would result in imbalanced exposure and fluctuation in the level, extent, and distribution of tissue injury in the colon between animals. These characteristics contribute to heterogeneity and restrict the ability to evaluate outcomes across studies conducted by various researchers [36].

The inflammatory colitis inducing agents are diluted in different levels of ethanol concentrations and administered via rectal instillation in the DNBS colitis models [37]. The ethanol treatment is required to interrupt the colonic mucosal barrier, enabling DNBS or TNBS to enter the lamina propria and haptenize the localised colonic and gut microbial proteins, allowing them to become immunogenic and stimulate host immune responses [38]. DNBS leads to severe inflammation in the colon and rectum, eliciting a strong inflammatory response associated with high levels of myeloperoxidase (MPO) [39], IL-1β and TNF-α [40]. Care must be taken during DNBS administration to ensure that the concentration of DNBS does not exceed to avoid rapid death of animals due to bowel punctures and sepsis. Due to the heterogeneity and inability to accurately monitor the degree of colitis in these chemically induce animal models of IBD, other models are required to study colitis in a more homogenous environment. As such the *Winnie* mouse model has been used to study colitis in animal models.

Mucin 2 is highly expressed on epithelial cells especially that of the colon. A single missense mutation in the *Muc2* mucin gene causes endoplasmic reticulum stress in intestinal goblet cells, a depleted intestinal mucus barrier, and spontaneous distal colonic inflammation by 6 weeks of age in the *Winnie* mouse model of spontaneous chronic colitis. [41]. *Winnie* mice exhibit symptoms of diarrhea, ulcerations and rectal bleeding and pain at different stages of colitis like human IBD [42]. Colitis in *Winnie* mice is initiated predominantly by an IL-23 mediated cytokine storm which can be ameliorated by administering anti-IL-23 monoclonal antibodies and dexamethasone [43]. As *Winnie* mice are created through a single nucleotide point mutation (which is not a gene deletion) causing clinical features resembling human colitis with an intact functional immune system, these mice are excellent pre-clinical models for studies in the pathophysiology of IBD. Colon cultures from *Winnie* mice, secrete high levels of IL-1β. Extensive studies undertaken in *Winnie’s* have rated them as the best murine model accessible for understanding human chronic colitis and its pathogenesis [42].

## 3. Inflammatory Mediators in Inflammatory Bowel Disease

Inflammation is induced because of tissue damage following an infection, chemical irritation, or shock [5]. In response to signals produced by resident macrophages, mast cells, or epithelium, neutrophils migrate to the site of inflammation [44,45,46]. A network of signalling molecules, including growth factors, cytokines, and chemokines, then recruits other immune cells to the site of inflammation [47,48]. During chronic inflammation, inflammatory infiltrates mainly consist of lymphocytes and macrophages [49]. Patients with IBD exhibit specific miRNA expression profiles [50], which might be involved in the initiation/development of inflammation [51,52,53,54,55,56]. Despite many miRNAs which contribute to the pathogenesis of IBD, the exact role of most is still unclear [57].

### 3.1. Immune Cells in Inflammatory Bowel Disease

Macrophages are the intermediary cells between the innate and adaptive immune systems, and are responsible for the secretion of growth factors, cytokines and reactive oxygen and nitrogen species [58,59,60,61]. Although these factors usually promote the inflammatory responses that lead to healing, prolonged inflammation may result in continuous tissue damage and subsequent sustained cell proliferation, potentially leading to malignant transformation [62,63,64,65,66]. Macrophages play a vital role in the pathogenesis of chronic inflammation and contribute to disease advancement and/or maintenance by secreting pro-inflammatory cytokines such as TNF-α [67], and are therefore, commonly linked with inflammatory-related diseases, including IBD.

**Figure 1:**
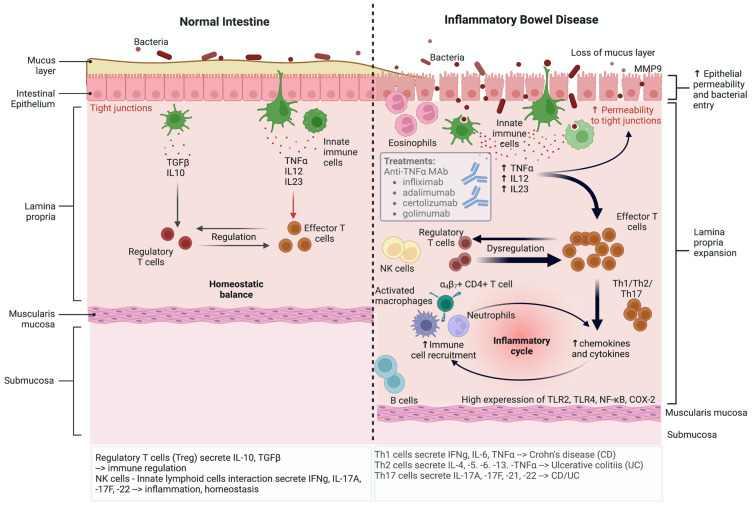
The immunological complexity of inflammatory bowel disease. CD, Crohn’s disease; COX-2, cyclooxygenase 2; IL, interleukin; IFN-γ, interferon gamma; MAb, monoclonal antibody; MMP9, matrix metallopeptidase 9; NF-κB, nuclear factor kappa-light-chain-enhancer of activated B cells; NK, natural killer; Th, helper T cells; TLR, Toll-like receptor; TNF-α, tumour necrosis factor alpha; UC, ulcerative colitis. Created using Biorender.com.

Eosinophils have also been associated with long-term intestinal inflammation and are abundantly present in IBD [68]. Eosinophils are not only involved in inflammation but also induce alterations to the enteric nervous system and are linked with disease severity [69]. The chemokine receptor, CCR3 which plays a role in the recruitment and activation of eosinophils has been shown that its blockade via a CCR3 antagonist attenuates disease severity and morphological damage to inflamed intestinal tissues in the spontaneous model of chronic colitis (*Winnie* mice) [70]. Similarly, in guinea pigs, intestinal inflammation/colitis caused following TNBS treatment results in increase of eosinophils at the site of inflammation. Using CCR3 antagonists alleviates enteric neuropathy and restores functional changes of the intestines [71]. Other immune cells have also been described in IBD and include dendritic cells, neutrophils, natural killer cells, T cells, B cells, Th1/Th17 cells, Th2 cells, regulatory T cells, leading to a complicated interaction between the immune cells, epithelial cells and the intestinal microbiota. Activation of the immune system highlights the role of immune cells in the pathophysiology of IBD (Figure 1) [72].

### 3.2. Cytokines and Chemokines

Cytokines control host immune response in infection, inflammation, and trauma. Proinflammatory cytokines, such as interleukin (IL)-1 which influences the tumour microenvironment and promotes cancer initiation and progression [73] and TNF-α which stimulates cancer cell growth, proliferation, invasion and metastasis, and tumour angiogenesis [15,74,75,76,77]. Chemokines, cytokines, and their downstream targets have received a lot of attention in the research on inflammation-induced cancer [5]. These inflammatory mediators promote tumour development, infiltration, metastasis and assist in angiogenesis [78]. The inflammatory cells, and their associated chemokines and cytokines affect the entire tumour organ and regulate the growth, migration and differentiation of all cell types including neoplastic cells, fibroblasts and endothelial cells in the TME [62,79,80]. Toll-like receptors (TLR) and nucleotide oligomerization domain receptors are pathogen-sensitive innate immune receptors that, when activated, cause the production of chemokines and cytokines that recruit immune cells [24]. TLR signalling pathways also stimulate production of other proinflammatory cytokines such as IL-12 and IL-6 [24]. Due to these effects, the function of cytokines in IBD which may lead to cancer is important to identify novel treatment of IBD.

TNF-α is one of the main pro-inflammatory cytokines, secreted by macrophages in IBD and has become a significant target for IBD therapy due to dramatic reduction of inflammatory markers and structural harm to the mucosa following its inhibition (Figure 1) [81,82]. The study of endogenous biochemical signals that attribute to chronic intestinal inflammation might lead to the development of more successful treatment [83,84]. Since TNF- functions have been found to be a major target for the development of treatments, the TNF-inflammatory pathway in IBD has been extensively studied. Several anti-TNF monoclonal antibodies, including infliximab, adalimumab, certolizumab pegol, and golimumab (Figure 1), have been developed because of this approach but these drugs are primarily ineffective in many patients or lose effectiveness over time [85]. However, the monoclonal antibody infliximab has been shown to effectively treat IBD [67].

### 3.3. Toll-Like Receptors

TLRs are transmembrane receptors, also known as pattern recognition receptors and stimulate pro/anti-inflammatory gene functions and restrict adaptive immune responses [24,86,87]. In CD, the regulation of TLR2 and TLR4 receptors are higher compared to healthy controls, which triggers faulty immune recognition [88,89], and is linked to chronic inflammation in IBD [24].

### 3.4. The Nuclear Factor Kappa B

The nuclear factor kappa B (NF-κB) pathway has been widely studied as a model of pro-inflammatory signalling pathway, due to its role in the expression of pro-inflammatory cytokines, chemokines, and adhesion molecules [90]. NF-κB regulates the expression of a variety of genes that are involved in the innate immune response, such as IL-1, IL-2, IL-6, IL-12, IFN-γ and TNF-*α* [24,91] and play key roles in many physiological and pathophysiological processes and mediating inflammatory signals [92]. In IBD these cytokines cause colonic tissue damage and NF-κB has been found to be a key regulator in this immune setting. NF-κB is over expressed in patients with IBD and influences mucosal inflammation [93]. Blockade of NF-κB activation has been used as a strategy to treat IBD. MG-341 an inhibitor of 26S proteasome, a target for NF-κB inhibition has been shown to attenuate colonic inflammation [93].

### 3.5. Matrix Metalloproteinases

Matrix metalloproteinases (MMPs), also known as matrixins, are very minimal or negligible in the usual tissues, however, their function is transcriptionally controlled by inflammatory cytokines, growth factors, hormones, as well as cell–cell and cell–matrix interactions [94]. MMPs are also regulated by precursor zymogen activation and prevented by endogenous inhibitors and tissue inhibitors of metalloproteinases (TIMPs) [95]. MMP-9 has been determined as a crucial pathogenic factor in IBD, being elevated in IBD patients exhibiting a malfunctioning intestinal tight-junction barrier with increased intestinal permeability [96]. The loss of intestinal epithelial barrier function is a significant factor in the onset and persistence of intestinal inflammation. (Figure 1). The specific role of MMP-9 in intestinal barrier function is still uncertain [97]. The inflammatory component of a developing neoplasm often includes a diverse leukocyte population—for example, neutrophils, dendritic cells, macrophages, eosinophils, mast cells, and lymphocytes - all of which secrete a wide array of cytokines and cytotoxic mediators including reactive oxygen species, serine, and cysteine proteases, MMPs, membrane-perforating agents, as well as soluble mediators of cell killing, such as IFNs, TNF-α, and cytokines [98,99].

### 3.6. Cyclooxygenase-2

COX-2, a key enzyme in fatty acid metabolism, is activated during both inflammation and cancer. It is stimulated by pro-inflammatory cytokines at the site of inflammation, and increased COX-2-induced prostaglandin synthesis facilitates cancer cell proliferation, angiogenesis, inhibits apoptosis, and enhances metastatic potential, making it a hot topic in research [100]. In IBD, COX-2 is highly induced by intestinal epithelial cells and since COX-2 plays a role in the development of CRC, inhibition of COX-2 may reduce the incidence of CRC. In fact, non-steroidal anti-inflammatory drugs and COX-inhibitors used in a double-blind placebo-controlled human clinical trial in patients with IBD was beneficial in most IBD patients without any exacerbations of IBD [101]. In addition, double targeting using an inhibitor of nitric oxide synthase as well as COX-2 inhibitor in IBD has the potential for the treatment of inflammation and colitis [102].

### 3.7. Myeloid Differentiation Primary Response 88

Myeloid differentiation primary response 88 (MyD88) is the established adaptor for inflammatory signalling pathways following activation of TLRs and IL-1 receptor families [103]. MyD88 signalling is involved in the advancement of CAC in colonic myeloid cells. Intestinal myeloid cells are important for maintaining local homeostasis and have a major role in regulating the existence of colitis and CAC [104]. MyD88 deletion also causes an increase in mucosal expression of COX-2, p-STAT3, β-catenin, and cyclinD1; all of which are associated with further DNA damage and β-catenin mutation. MyD88 knockout mice develop severe colitis and macrophage and CD4+ T cell infiltration in the intestinal mucosa, following the addition of dextran sulfate sodium in the drinking water [105]. In addition, MyD88 knockout mice infected with *Salmonella Typhimurium* endured enhanced intestinal tissue loss and showed barrier disruption, compared to wild-type mice [106]. Thus, myeloid MyD88 signalling protects the intestine from inflammation as well as tumourigenesis during the development of CAC [104].

## 4. Inflammation and Cancer

There is a firm link between chronic inflammation and cancer, mediated by several inflammatory pathways including cytokines and mediators. In addition to exogenous mutagens, immune cell infiltrates in the colon develop an environment rich in reactive oxygen and nitrogen species, which can result in DNA damage, enabling the onset of oncogenesis [107]. Dysplasia can be polypoid or plain, localised, distributed, or multifocal in IBD patients. When dysplasia is observed in patients, it exposes the entire colon at risk of neoplasia, requiring surgical removal of the entire colon and rectum. These morphological and biological differences pose major challenges in clinical cancer surveillance in IBD patients more than in the general population, raising critical questions over chronic inflammation’s contribution to the develop CRC.

### 4.1. Colitis Associated Cancer/Colorectal Cancer

CAC is a subset of CRC that can be developed in patients with long-standing IBD. The role of inflammation as a risk factor in CRC has been largely investigated, both in CAC and sporadic tumours [108]. CRC is the third most common malignancy in the world, with more than 1 million annual new cases worldwide [109]. CRC is linked to prolonged period of colitis and anatomic significance, along with the presence of other inflammatory symptoms such as primary sclerosing cholangitis [110]. Colitis-associated CRC and random CRC are different in terms of presentation and molecular characteristics. These variations are brought on by variations in DNA methylation, which induces changes in gene expression [111]. The use of drugs for treating inflammation, such as 5-aminosalicylates and steroids, may inhibit the advancement of CRC in IBD [112]. CRC affects the caecum, colon, and rectum; one of the most diagnosed and causes of cancer deaths worldwide. Most CRC patients receive chemotherapy prior to or following surgery, however, diarrhea, constipation, oral mucositis, nausea, and vomiting are frequent gastrointestinal side effects occurring in up to 80–90% of patients. These side effects may also result in the development of malnutrition and dehydration in patients leading to rapid weight loss (cachexia) [113]. Early death rates of up to 4.8% associated with chemotherapy are due to gastrointestinal toxicity [114]. Due to these complications, the administration of chemotherapy is often restricted, leading to reduction in efficacy. Although animal models of IBD have given important insights into the underlying cause of CAC, the molecular processes by which inflammation stimulates cancer remain poorly understood [115].

### 4.2. Cancer Microenvironment

**Figure 2:**
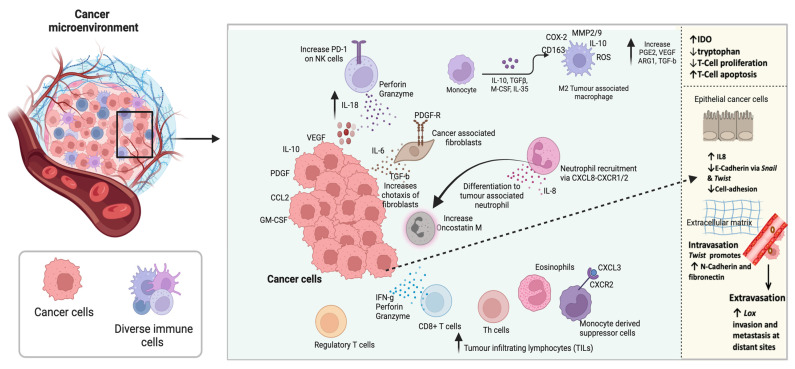
The immunological complexity of the cancer microenvironment. Created using Biorender.com. ARG1, arginase 1; CCL2, C-C motif chemokine ligand 2; CD, cluster differentiation; COX-2, cyclooxygenase 2; CXCL3, chemokine (C-X-C motif) ligand 3; CXCR2, C-X-C motif chemokine receptor 2; GM-CSF, granulocyte macrophage colony stimulating factor; IDO, indoleamine-2,3-dioxygenase 1; IL, interleukin; Lox, lysyl oxidase; M-CSF, macrophage colony stimulating factor; MMP2/9, matrix metallopeptidase 2/9; NK, natural killer; PD-1, programmed cell death protein 1; PDE2, phosphodiesterase 2; PDGF, platelet derived growth factor; PD-L1, programmed death ligand 1; ROS, reactive oxygen species; TGFb, tumour growth factor beta; VEGF, vascular endothelial growth factor.

The cancer microenvironment refers to cells present around the cancer cells which include, fibroblasts, natural killer cells, macrophages, monocyte derived dendritic cells, CD4+ T cells, CD8+ T cells, regulatory T cells, eosinophils, neutrophils, blood vessels and proteins produced by all these cells and support the development of cancer cells [116]. The cancer microenvironment is quite complex (Figure 2) [15,117]. The range of innate and adaptive immune cells present in the cancer microenvironment secrete both pro- and anti-tumourigenic mediators. Cancer cell interactions with its surrounding microenvironment (stromal cells, extracellular matrix, immune cells), are important for cancer cells clonal evolution, heterogeneity, as well as developing resistance against drugs, which all lead to the proliferation, growth and metastasis of cancer cells [118]. In addition, the nervous system interacts with cancer cells, adding to their regulation, growth, angiogenesis, and metastasis [74,75,76]. Cancer immunotherapy involves boosting of immune CD4+, CD8+ T cells and B cells to kill or block cancer cells [119,120]. However, cancer cells have evolved, and certain checkpoint makers are expressed which allows their escape from immune attack. These include cytotoxic T-lymphocyte-associated antigen (CTLA-4) and programmed cell death (PD-1) or programmed cell death ligand 1 (PD-L1). The PD-1/PD-L1 pathway is the frontline of interactions between immune cells, stromal cells, and cancer cells [121].

## 5. Checkpoint Markers

**Figure 3:**
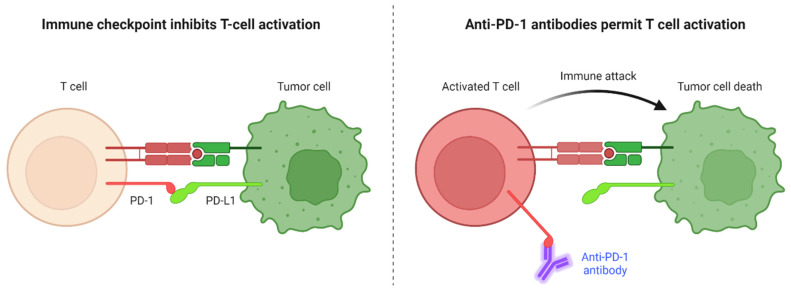
Immune checkpoints inhibit T cell activation in the cancer microenvironment, upon ligation of programmed cell death protein 1 (PD-1) expressed by T cells to its ligand programmed death ligand 1 (PD-L1) on cancer cells. Anti-PD-1 monoclonal antibodies interfere with PD-1/PD-L1 interaction and permits T cell activation and lysis of cancer cells. Figure created using Biorender.com.

The ability of the immune system to distinguish between self and non-self-antigens using “checkpoints” is one of its most important functions. Checkpoint markers are present on cancer cells as a means of evading immune attack. Checkpoint inhibitors therapy differ from traditional chemotherapy by increasing the activation of immune cells, specifically T cells. (Figure 3) [16]. In contrast to chemotherapy, tolerance to checkpoint inhibitors appears to be higher, resulting in fewer side effects and a better outcome for cancer patients [122]. ICIs are monoclonal antibodies designated for an increasing number of malignant diseases. Checkpoint inhibitors include cytotoxic T-lymphocyte-associated antigen (CTLA)-4 inhibitors (ipilimumab, tremelimumab) and programmed cell death protein 1 pathway/programmed cell death protein 1 ligand inhibitors (PD1/PD-L1) (pembrolizumab, nivolumab, durvalumab, atezolizumab), are more frequently used in clinical trials for the treatment of several cancers [123]. Determining the role of PD-1/PD-L1, PD-L2, indoleamine 2,3-dioxygenase (IDO), sialic acid-binding immunoglobulin-like lectins (Siglecs), and CTLA-4 in animal models of IBD, patients with IBD, as well as in CRC can provide insights into disease initiation and progression that may assist in identification of novel targets. Furthermore, anti-PD-1/PD-L1 therapies are not relevant to all patients, implying the need to identify additional targetable immune checkpoints [124].

### 5.1. Role of Checkpoint Inhibitors

The purpose of cancer immunotherapy is to stimulate cytotoxic T lymphocytes/CD8+ T cells against tumour associated proteins/receptors and, help the initiation of tumour specific T cells in lymphoid organs to achieve efficient and long-lasting anti-tumour immunity [125]. However, the TME is complicated involving immune cells, cytokines, and checkpoint markers [121]. Using ICIs as a single agent or in combination treatments with chemotherapy, radiotherapy, or immunotherapeutic intervention, have produced efficient and long-term clinical outcomes [19].

PD-1 (CD279) is a checkpoint molecule on T cells that prevent T cells from damaging its own cells in the body. It is present on activated T cells and binds to PD-L1 or PD-L2 on tumour cells, causing deactivation and death of T cells. In mouse models, the loss of PD-1 expression on T cells has been observed to substantially prolong tumour growth and increase CD8+ T cells inside the TME (Figure 3). Nivolumab, is a PD-1 inhibitor, approved by the FDA is in use for metastatic melanoma [126]. In mouse models of IBD and IBD patients, PD-1 is upregulated on T cells, macrophages, dendritic cells, B cells and in colon tissues that are inflamed. Some examples of drugs that target PD-1 include Pembrolizumab (Keytruda) and Nivolumab [127]. In addition, ipilimumab (Yervoy) is a monoclonal antibody that inhibits CTLA-4 activity, providing a similar effect. Drugs that target the PD-1 and CTLA-4 pathways have specifically demonstrated considerable clinical efficiency and gained approval as single-agent or combination therapy for regular use [128]. Advances in immuno-oncology have started to revolutionize the standard of care for many types of cancer. Pembrolizumab and nivolumab are two PD1-blocking antibodies that have shown effectiveness in individuals with metastatic CRC (mismatch-repair-deficient) and microsatellite instability-high (dMMR-MSI-H) and have gained rapid FDA approval [129].

**Figure 4:**
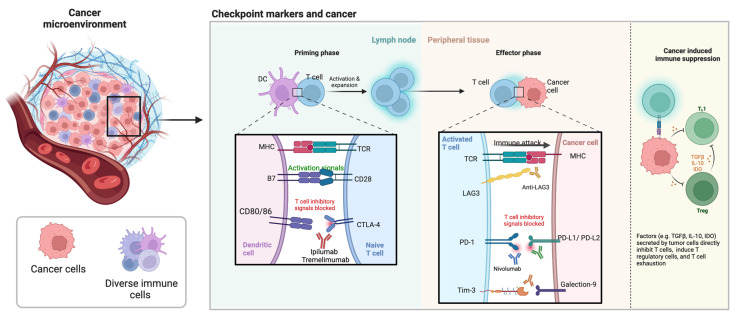
The cancer microenvironment is complex involving diverse immune cells and checkpoint markers. Figure reproduced from Ephraim et al. [121]. CD, cluster of differentiation; CTLA-4, cytotoxic T lymphocyte associated protein 4; DC, dendritic cells; IDO, indoleamine 2,3-dioxygenase; IL, interleukin; LAG3, lymphocyte activation gene 3; MHC, major histocompatibility complex; PD-1, programmed cell death protein 1; PDL-1, programmed death ligand 1; PDL-2, programmed death ligand 2; TCR, T cell receptor; TGF, tumour necrosis factor; Tim-3, T cell and immunoglobulin mucin domain 3; Treg, regulatory T cells.

CTLA-4, also known as CD152, is an immune checkpoint receptor that inhibits CD8+ T-cell activation. It is present on regulatory T cells and facilitates their immunosuppressive effect (Figure 4) [130]. Anti-CTLA-4 monoclonal antibodies—ipilimumab and tremelimumab can prevent CTLA-4 ligand-driven immunosuppression [131]. CTLA-4 and PD-1 are inhibitors of T-cell immune function [132]. CTLA-4 is a CD28 homolog has a stronger bond with B7 [132]. CTLA4 is a candidate gene that has been linked to the progression of CRC [133].

Sialic acid-binding immunoglobulin-like lectins (Siglecs) are transmembrane sialic acid-binding proteins of the immunoglobulin superfamily that contain an N-terminal V-set Ig-like domain as well as a variable number of C2 set domains. (Table 1) [134]. Siglec-3 is also known as CD33 and has been determined as a myeloid lymphoma marker in clinical studies much before any other Siglec was discovered [135]. Siglec-3 was mapped to chromosome 19 and previous studies on cDNA isolation and cloning suggested CD33 association with MAG (Siglec-4) [136]. Siglec-8 is present on the surface of human eosinophils, mast cells, and basophils, and its activation by specific glycan ligands or antibodies initiates loss of eosinophils and reduces mast cell degranulation [137]. Siglec-8 promotes cytokine-dependent death [138]. Siglec-9 is upregulated on neutrophils and induces cell death when associated with monoclonal antibodies [139]. Current research on Siglecs have demonstrated major roles in tumour immunosurveillance including immunosuppression, that are appealing anti-cancer molecular targets [124].

Indoleamine 2,3-dioxygenase (IDO) is an enzyme that reduces tryptophan, an essential amino acid [156], and is overexpressed in the colon, intestines as well as the lung. The mechanism by which IDO works is by catalysing the oxidative ring cleavage of pyrrole’s in tryptophan, serotonin, melatonin, and other indoleamine derivatives [157]. In CRC, the regulation of IDO by tumours is associated with metastases and inversely linked with infiltration of T cells [158]. The expression of IDO1 could be generated by IFN-γ, lipopolysaccharide, and tumour necrosis factor (TNF). Thus, in response to inflammatory signals under pathophysiological conditions IDO1 is highly up regulated by the immune system, and over-expression of IDO1 improves detection in different types of cancers, including melanoma, pancreatic, ovarian, and colorectal [159]. The IDO1 paralog IDO2, despite having received far less research, may be a possible alternative as a therapeutic target in cancer immunotherapy. IDO2 is substantially less efficient than IDO1 at metabolising tryptophan, and its functions are instead the result of interactions with other, as-yet-unidentified proteins that may change in various inflammatory and neoplastic circumstances [160].

T cell immunoglobulin and mucin domain-containing protein 3 (Tim-3) is a marker selectively present on IFN-γ–producing CD4+ T helper 1 (Th1) and CD8+ T cytotoxic 1 (Tc1) T cells (Figure 4) [161]. Tim-3 is upregulated in CRC, compared to normal tissues [162]. The Tim-3 pathway is a target for anticancer immunotherapy due to its expression on both non-functional CD8^+^ T cells and Tregs—two main immune cell groups that suppress the immune activity in tumour tissue [163] and has exhibited remarkable results in preclinical cancer models. Tim-3/PD-1 pathway co-blockade is more successful than either Tim-3 or PD-1 pathway blockade only at rebuilding tumour antigen–specific IFN-γ production in CD8^+^ T cells in mice carrying tumours [164].

Lymphocyte-activation gene 3 (LAG-3) is a critical immune checkpoint marker with implications for a variety of diseases, including cancer. (Figure 4) [165]. LAG-3, along with other ligands such as galectin-3 and LSEC-tin, binds to MHC class II. LAG3 (or CD223), like PD-1, is expressed on a variety of cell types, such as tumor-infiltrating lymphocytes (CD4, CD8) and regulatory T cells. LAG3 is essential for effective T cell expression and homeostasis [166]. Suppression of LAG3 showed reduced tumour growth which was not very effective, however, suppression of LAG3 and PD-1 together, not only reduced tumour growth but also increased survival rate in mice [167,168]. Tumour-infiltrating lymphocytes expressing elevated levels of LAG3 have been found in solid tumours such as ovarian cancer, melanoma, and colorectal cancer, as well as Hodgkin’s and diffuse large B-cell lymphoma [169]. LAG3 inhibits cytokine and granzyme production and proliferation while promoting regulatory T cell differentiation [165].

### 5.2. Stimulatory Checkpoint Molecules

The CD70-CD27 axis promotes T-lymphocyte expansion and differentiation by stimulating the NF-B pathway [170]. CD28 is present on almost all human CD4+ T cells and most ofCD8+ T cells. Binding with its two ligands -CD80 and CD86, present on dendritic cells, initiates T cell expansion [171]. CD40 and its ligand CD40L may trigger antigen presenting cells to permit CD8+ T cell priming [172]. CD40 is a key signalling pathway for the function of B cells, monocytes, and dendritic cells, and plays an important role in the inflammatory pathways of non-hemopoietic cells. CD40 is expressed by monocytes and dendritic cells, and overexpressed when dendritic cells in response to microbial threat, cells move from the periphery to depleting lymph nodes [173]. CD40 protein expression is significantly higher in CRC compared with normal tissue [174]. The first proof-of-concept study that shows how using CD122 alone or in conjunction with a vaccination or a Glucocorticoid-induced TNFR-related protein (GITR) monoclonal antibody immunotherapy might boost and maintain anticancer responses. These findings support the use of CD122 as a monotherapy target or in combination with other immune-targeted treatments for CRC [175]. CD137, an inducible T-cell costimulatory receptor and a member of the TNF receptor superfamily. CD137 and CD137L expression was upregulated in all investigated colon cancer tissues compared to normal colon tissues. Targeted microenvironment imaging strategies may be used to facilitate early detection of tumours, and isotope labelled anti-CD-11b could be of further evaluated as a potential probe [176]. Inflammatory responses closely regulate CD163 expression, with anti-inflammatory signals (e.g., IL-10, glucocorticoids) stimulating CD163 expression while proinflammatory signals (e.g., lipopolysaccharide, TNF-α, IFN-γ) suppressing CD163 synthesis [177].

The B7 ligand family comprises of 10 members -CD80 (B7-1), CD86 (B7-2), PD-L1 (B7-H1), PD-L2 (B7-DC or CD273), ICOSL (B7-H2), CD276 (B7-H3), B7S1 (B7-H4, B7x or Vtcn1), VISTA (B7-H5, GI24, or PD-1H), B7-H6, and B7-H7 (HHLA2) [178]. B7-1 or CD80 is upregulated in dysplastic colonic mucosa of UC patients, with CD80 signalling between intestinal epithelial cells and T-cells representing a critical factor in the development of inflammatory colonic carcinogenesis from low to advanced dysplasia [179]. Oncogenic insults, such as oxidative DNA damage linked to long-term intestinal inflammation, can induce CD80 expression. The stromal B7-2 or CD86/CD163 ratio could be used for personal risk assessment of relapse and mortality for stage II-III CRC [180]. Together with tumour staging, this ratio may aid in personalized treatment. B7-H1, PD-L1, or CD274 are upregulated in colorectal carcinoma and have been linked to cell differentiation and tumor-node-metastasis placement [181]. B7-H2 or Inducible costimulatory ligand (ICOS-L) expressed on CD8+ T cells in the tumour micro-environment are closely associated with progression of CRC [182]. B7-H3 or CD276 are potentially associated with CRC advancement and evasion of cancer immune surveillance [183]. B7-H4 is associated with CD133 and CD44 regulation in CRC tissues, and B7-H4 knockout mice prevents growth of tumour spheroids, cell migration, and infiltration in CRC cell lines which shows that it could be a potential prognostic biomarker for CRC [184]. B7-H5 (also known as V-domain immunoglobulin suppressor of T cell activation (VISTA), C10orf54, PD-1H, Gi24, and Dies1) is expressed at higher levels in cancer sections compared to non-cancer tissues, promotes tumour immune escape, associated with lymph node participation, cancer stage, and survival [185]. As such, B7-H5 is an important marker for prognosis and a potential target for the immunotherapy of CRC. B7-H6 is activated at the surface of CD14^+^CD16^+^ proinflammatory monocytes and neutrophils in response to TLR or pro-inflammatory cytokines such as IL-1β and TNFα [186]. The novel B7-H6/CD3 bispecific IgG-like T cell retaining antibody for the treatment of CRC and haematologic malignant cells [187]. The level of HHLA2 expression in CRC patients was discovered to be positively connected with a high death rate and strongly associated with the complexity of invasion and CD8+ T cell infiltration status, implying that it may operate as an independent prognostic factor related with overall survival; however, the thorough regulation of HHLA2 in CRC remains unclear. Human leukocyte antigen (HLA)-G, a member of the HLA family, serum sHLA-G levels correspond with disease severity in paediatric CD patients and are higher in CD patients than in UC patients. As a result, sHLA-G may be a biomarker for disease severity in CD [188]. In both haematological and solid tumours, there is a high rate of HLA-G surface activity and elevated sHLA-G serum levels. HLA-G and sHLA-G regulation is associated with poor treatment results in cancer patients, implying a role in cancer cells’ immune escape mechanism [189].

## 6. Challenges and Gaps

Although the frequency of checkpoint inhibitors cause adverse events, it is much less than chemotherapy treatments, their use may lead to emergency ward if severe immune-related adverse events (irAEs). The occurrence of irAEs is also less frequent following immunotherapy compared to chemotherapy, however, its best that intensivists are familiar with the side effects of these medications, especially if in intensive care unit admission is required [123]. The immunosuppressive features of tumour lesions play a big part in the advancement of cancer and are a big challenge for effective immunotherapies. Blocking the pathways of PD-1/PD-L1 has demonstrated excellent therapeutic efficacy for cancer patients with a variety of diseases. However, the effectiveness of authorised ICIs in treating CRC is poor, and only a small proportion of individuals benefit from them. The current ICIs are not suitable for most CRC patients with microsatellite stable or mismatch repair proficiency or reduced levels of microsatellite instability. This inadequate treatment effectiveness highlights the critical need to identify more checkpoint markers in CRC. 

## 7. Conclusions and Future Prospects

Though there has been significant research and information on checkpoint markers, their roles in IBD and CRC development remain unclear and require further study. Studying checkpoint markers in the Winnie mouse models that closely resemble human models may lead to the discovery of biomarkers for screening IBD patients and for the advancement of understanding inflammation and cancer that may lead to designing drugs that use inhibitors of biomarker expression or use vaccines to prevent disease progression. However, these treatments require significant improvement, one of the main aspectss is to identify the “baseline (pre-treatment)” biomarkers to predict immune responses. In general, biomarkers are mainly divided into two functional categories: “prognostic” and “predictive” [190]. A prognostic biomarker can be defined based on the way patient’s body or tumour biology influence the patient’s clinical outcome. This includes patients at high risk for disease relapse who may benefit from earlier treatments. On the other hand, a predictive biomarker is defined by the effects of treatment, including tumour response, and the improvements in overall survival of patients, disease-free survival, and progression-free survival [191]. Further research into these animal models and studying the function and effects of ICIs in these animal models will shed light on new targeted therapies for IBD as well as CRC.

## Figures and Tables

**Table 1:** Sialic acid-binding immunoglobulin-like lectins (Siglecs) and their function.

Siglecs	Function	References
Siglec-1 (CD169)/Sialoadhesin	Cell adhesion, cancer progression	[140,141]
Siglec-2 (CD22)	Dampening B cell receptor activation	[142]
Siglec-3 (CD33)	Downstream signalling function	[143]
Siglec-4 (Myelin Associated Glycoprotein, MAG)	Interaction between MAG and cancer-associated MUC1, stabilizes myelin-axon interactions	[124,144]
Siglec-5 (CD170)	Associates with leukocyte counter-receptor P-selectin glycoprotein ligand-1, prevent leukocyte recruitment to sites of inflammation and maintains a pro-cancer environment.	[145]
Siglec-6 (CD327)	Immune-inhibitory, inhibitory receptor on mast cells in CRC	[146]
Siglec-7 (p75/AIRM1, CD328)	Natural killer (NK) cell-inhibitory receptor bearing immunoreceptor tyrosine-based inhibition (ITIM) motifs	[147]
Siglec-8	A target in allergen-induced inflammation	[148]
Siglec-9 (CD329)	Binds to MUC1 with sialylated T-antigen (MUC1 ST)	[149]
Siglec-10	Repress DAMP-mediated innate inflammatory responses	[150]
Siglec-11	Inhibitory function, microglial activities	[124,151]
Siglec-12 (Siglec-XII)	Recruit SHP2-related oncogenic pathways	[152]
Siglec-13	Deleted in humans	[124]
Siglec-14	Suppresses myeloid inflammatory responses	[153]
Siglec-15	Upregulated on cancer cells and tumour-infiltrating myeloid cells	[154]
Siglec-16	Expressed on cancer cells	[155]
Siglec-17	Deleted in humans but pseudogene exists	[124]
